# Which AI doctor would you like to see? Emulating healthcare provider–patient communication models with GPT-4: proof-of-concept and ethical exploration

**DOI:** 10.1136/jme-2024-110256

**Published:** 2026-05-05

**Authors:** Hazem Zohny, Jemima Winifred Allen, Dominic Wilkinson, Julian Savulescu

**Affiliations:** 1 Oxford Uehiro Centre for Practical Ethics, Oxford University, Oxford, UK; 2 Philosophy, University of Oxford Uehiro Centre for Practical Ethics, Oxford, UK; 3 Department of Paediatrics, Monash University, Melbourne, Victoria, Australia; 4 Oxford University Hospitals NHS Foundation Trust, Oxford, UK; 5 Centre for Biomedical Ethics, Yong Loo Lin School of Medicine, National University of Singapore, Singapore; 6 Wellcome Centre for Ethics and Humanities, Oxford University, Oxford, UK

## Abstract

Large language models (LLMs) have demonstrated potential in enhancing various aspects of healthcare, including health provider–patient communication. However, some have raised the concern that such communication may adopt implicit communication norms that deviate from what patients want or need from talking with their healthcare provider. This paper explores the possibility of using LLMs to enable patients to choose their preferred communication style when discussing their medical cases. By providing a proof-of-concept demonstration using ChatGPT-4, we suggest LLMs can emulate different healthcare provider–patient communication approaches (building on Emanuel and Emanuel's four models: paternalistic, informative, interpretive and deliberative). This allows patients to engage in a communication style that aligns with their individual needs and preferences. We also highlight potential risks associated with using LLMs in healthcare communication, such as reinforcing patients_’_ biases and the persuasive capabilities of LLMs that may lead to unintended manipulation.

## Introduction

Large language models (LLMs) show promise in enhancing medical decision-making, healthcare provider (HCP)–patient communication and clinical reasoning. For instance, LLMs have demonstrated potential in healthcare by generating higher-quality, more empathetic responses to patient questions on Reddit’s r/AskDocs forum compared with physician responses,^1^ as well as compared with offline, real-world physician–patient interactions,^2^ and more specifically to cancer-related questions compared with licensed physicians.^3^ There are also indications that they surpass doctoral students in social intelligence measures,^4^ outperform physicians in clinical reasoning documentation quality,^5^ accurately assess patient condition severity in emergency departments, may potentially improve the triage process^6^ and enhance informed decision-making in the consent process for medical procedures.^7^ These studies, while subject to numerous caveats and limitations, are indicative of a trend to seriously evaluate the potential for deploying LLMs in different aspects of healthcare.

When it comes to communication with patients specifically, some have raised a concern that artificial intelligence (AI) systems more generally may adopt implicit communication norms that deviate from what patients want or need from talking with their doctor. If AI fails to consider a patient_’_s values, it threatens to reintroduce paternalism, potentially hindering patient autonomy and shared decision-making in doctor–patient relationships.^8–10^

For example, Savulescu *et al*^8^ note that IBM_’_s Watson for Oncology system ranks treatments according to length of life improvements rather than quality of life and does not encourage recognising treatment decisions as value laden. For that reason, they argue that doctors must be involved in translating AI recommendations to patient-centred care to avoid this ‘machine paternalism.’ These concerns echo the argument that medical AI systems should be designed to be value flexible, accommodating diverse patient values and facilitating shared decision-making between doctors and patients.^11^

Building on these initial explorations, and partly in response to concerns about machine paternalism,^8^ this paper investigates a further potential application of LLMs in healthcare: empowering patients to discuss and understand their own medical cases through their preferred communication styles. Specifically, we explore a potential role of LLMs in providing patients with a platform to understand their medical conditions and express their concerns, preferences and values in a manner that aligns with their desired medical communication approach.

The context for this article is the longstanding debate surrounding the appropriate communication style that HCPs should employ when discussing medical conditions with their patients. Emanuel and Emanuel^12^ influentially proposed four distinct models: paternalistic, informative, interpretive and deliberative, each with its own strengths and weaknesses. For instance, while the paternalistic approach may be suitable in some emergency situations, in other contexts it places insufficient emphasis on patient autonomy. Conversely, the deliberative approach emphasises collaborative decision-making but requires significantly more time, skills and resources.

Recognising that no single communication style is ideal for all patients and situations, one potential solution is to empower patients to choose their preferred communication style based on their individual needs, preferences and values.^13^ However, patients may not always know which model best suits them, and HCPs may lack the time or ability to seamlessly switch between different relationship models depending on patient preference. Moreover, the same patient may benefit from different communication styles throughout their treatment process, as clinical circumstances and patient information need change. It is in this context that we wish to explore the potential for LLMs to play a role in helping patients explore their values, understand their medical conditions, and shape the content and form of healthcare communication.

To begin, we first elaborate Emanuel and Emanuel’s commonly cited taxonomy of HCP–patient relationship styles.^12^ Then, we provide a proof-of-concept demonstration using ChatGPT-4, showcasing how an LLM can be instructed to emulate each of the four relationship dynamics described by Emanuel and Emanuel. This is then followed by a discussion of the potential benefits, risks, and future research directions.

### The Dilemma Over ‘The Right’ Communication Style

The history of Western HCP–patient relationships can be broadly characterised as shifting from one of paternalism, to one of focusing on patient autonomy and increasing patient involvement in shared decision-making.^14^ Exactly what patient autonomy amounts to in the face of clinician’s medical expertise is contentious, and there is disagreement regarding how to distinguish patient-centred care from models on which patients are understood as mere consumers.^15^ One way of understanding patient autonomy in the context of a relationship with a provider is by contrasting different models of HCP communication.

Emanuel and Emanuel described four such models.^12^ We elaborate on them here as their details are relevant to understanding the key characteristics of each approach and how they are reflected in attempts to emulate them by GPT-4.

The paternalistic approach entrusts providers with the obligation to advance patients’ interests. Patients’ preferences are trumped by the physician’s judgement concerning the patient’s best interests. An opposing approach is the ‘informative’ approach, which positions the provider as a transmitter of pertinent factual data, enabling the patient to use (or disregard) the information to choose an intervention based on their preferences.

A third model, known as the interpretive approach, requires the provider not only to relay factual information but also aid the patient in uncovering and interpreting their own values in light of the provided data, facilitating an informed decision congruent with the patient’s newfound self-awareness. Values-based practice could be characterised as an example of this model in which HCPs support clinical decision-making by eliciting and integrating patients’ individual values.^16^

Lastly, the deliberative approach expands on the informative and interpretive models by assigning the provider a role analogous to that of a friend or educator, engaging the patient in normative dialogue and, when appropriate, actively endeavouring to persuade (without coercing) the patient towards the most suitable course of action following the interpretive process. An approximate example of this is the liberal rationalist model, which emphasises rational dialogue between HCPs and patients, focusing on an objective, shared understanding of what is good for the patient.^17 18^

These by no means exhaust the possible relationship dynamics between HCPs and patients, and the appropriate model may vary depending on the medical context. In emergencies, immediate intervention could take precedence over patient value clarification. Moreover, patients themselves vary in their preferences for the type of relationship they desire with HCPs.^19^ Some patients may strongly prefer a collaborative or deliberative approach, wanting to engage in extensive dialogue about their values and treatment options. They may wish to take an active role in decision-making and have their autonomy respected to the greatest extent possible.

On the other hand, some patients may prefer a more directive or informative approach from their providers. They may feel overwhelmed by the complexity of medical information and decisions and desire clear guidance from an expert they trust.^20^ Cultural factors, personality traits and the emotional impact of illness can all influence these preferences.^21^

Patients also vary significantly in their health literacy and ability to comprehend complex medical concepts.^22^ Those with lower health literacy may require much more extensive discussion and clarification from their HCPs to feel adequately informed and empowered in their decision-making. They may benefit from longer appointments, the use of simple language and visual aids, and the opportunity to ask questions and receive explanations multiple times. Conversely, patients with high health literacy may feel frustrated by overly simplistic explanations and prefer efficient, data-driven conversations. Linked to this, HCPs may perpetuate disparities by making assumptions about patients’ health literacy, leading to inconsistent information sharing.

While contemporary literature emphasises shared decision-making and variations of the interpretive and deliberative approaches, these models still pose challenges. HCPs may inadvertently impose their own values onto patients, especially when patients are overwhelmed or uncertain about their values.^12 23^ This is particularly relevant to the deliberative model, where patients may struggle to respond to HCPs advocating a specific course of action, even if the final decision lies with the patient. Some patients might avoid seeking healthcare altogether if they anticipate efforts to persuade them towards a treatment they resist. Moreover, it is difficult to agree on what constitutes ‘too much’ or even the right kind of persuasion in a patient-centred framework.^13^

One strategy in response to such concerns is to allow patients to select their preferred relationship dynamic with their HCP.^13^ This could be achieved via an initial conversation between HCPs and patients to determine the preferred relationship dynamic and the degree of persuasion a patient is willing to be subjected to.

However, patients may not know which model they prefer, and even if they did, HCPs may not have the time or skills to deploy the particular relationship dynamics that a patient prefers.

This is where LLMs may play a role. By engaging patients in exploratory conversations and presenting them with information about the different relationship models, LLMs could help patients gain clarity about their preferences and make more informed decisions about their preferred communication style with their HCPs. More radically, LLMs could become the primary tool for value exploration and elucidation, particularly for patients who wish to engage with their medical condition in-depth. The following section illustrates this potential role of LLMs in more detail and provides a proof-of-concept demonstration using ChatGPT-4.

### Medical Communication-Style Emulator: A Proof Of Concept

LLMs use deep learning techniques to identify patterns within large text datasets, allowing them to generate human-like, contextually relevant responses.^24^ During training, LLMs process each token—basic units of text that may be parts of words or whole words—in relation to the tokens that appear before and after it in a sentence. This allows the model to learn how words are typically used in context.^25^ For example, the token ‘cold’ can refer to a common illness or a sensation of low temperature, and the model learns to distinguish between these meanings based on the surrounding tokens. LLMs extend this contextual understanding to recognise complex linguistic patterns, including tone and style. This enables them to adapt to various communication styles when given examples or instructions.^26^

In this section, we present illustrative examples of how LLMs can emulate the four HCP–patient relationship models described by Emanuel and Emanuel^12^: paternalistic, informative, interpretive and deliberative. Each dialogue, generated using GPT-4, showcases the model’s ability to engage in meaningful and contextually appropriate interactions based on the patient’s clinical situation and expressed values.

Table 1 includes extracts from longer dialogues (see online supplemental materials) based on a hypothetical case presented in Emanuel and Emanuel’s paper. It involves a 43-year-old premenopausal woman who has been diagnosed with a 3.5-cm ductal carcinoma of the breast that is oestrogen receptor positive.^12^ These brief details were included in the prompts that triggered these dialogues.

To generate the dialogues, we provided the same patient case to each prompt and instructed GPT-4 to generate a conversation that epitomised one of the four HCP-patient models. While GPT-4 demonstrated familiarity with Emanuel and Emanuel’s four models, we included the exact description of each communication model in the prompt to maximise consistency. Readers can read the full exchange in the supplementary materials, but to illustrate, here is the prompt used for the deliberative dialogue, which includes a direct quote from the Emanuel and Emanuel (p2222):

Create a dialogue between a patient with the attached case [Emanuel and Emanuel’s breast cancer case], and a doctor who is deploying the deliberative approach. Remember, on the deliberative model, the aim of the physician-patient interaction is to help the patient determine and choose the best health-related values that can be realized in the clinical situation. To this end, the physician must delineate information on the patient’s clinical situation and then help elucidate the types of values embodied in the available options. The physician’s objectives include suggesting why certain health related values are more worthy and should be aspired to. At the extreme, the physician and patient engage in deliberation about what kind of health-related values the patient could and ultimately should pursue.

To analyse the transcripts, we used the descriptions provided in Emanuel and Emanuel’s paper to identify the key characteristics of each approach. Table 1 includes exchanges that exemplify how GPT-4 embodied these characteristics in its responses.

Readers can see how, in the paternalistic dialogue, GPT-4 provided decisive information and recommended a specific treatment plan (P1 and P2), using persuasive language to convince the patient of the recommended course of action (P3). It addressed the patient’s concerns with authority and emphasised the importance of prompt action (P4 and P5). In contrast, the informative model focused on providing detailed information about the diagnosis, treatment options, risks and benefits (INF1, INF2 and INF3), addressing the patient’s specific concerns by offering additional information and explanations (INF4). GPT-4 facilitated decision-making by encouraging the patient to weigh the pros and cons of each option based on their personal preferences and lifestyle (INF 5).

The interpretive and deliberative models demonstrated a more collaborative approach. In the interpretive model, GPT-4 sought to understand the patient’s values, priorities and concerns (INT1), helping them balance treatment options with their values and priorities (INT2). It elucidated and clarified the patient’s values to help them make an informed decision (INT3), formulating a treatment plan based on the patient’s expressed values (INT4) and ensuring alignment between the treatment plan and the patient’s life goals (INT5). Similarly, the deliberative model explored the patient’s values and helped them determine which health-related values are most important (D1). It discussed treatment options and their implications in relation to the patient’s values (D2), balancing the patient’s values with treatment efficacy to help them make an informed decision (D3). GPT-4 aligned the treatment plan with the patient’s expressed values (D4) and engaged in collaborative decision-making, ensuring that the chosen plan aligns with the patient’s values and priorities (D5).

While this was designed as a proof of concept, the dialogues generated by GPT-4 reflect its potential to adapt to different communication styles. They provide a starting point for further exploration and analysis of how LLMs might be used to enable patients to choose their preferred communication style when considering their own medical cases.

Nonetheless, there are a number of obvious limitations. First, the illustrative examples generated by GPT-4 may not be representative of all possible outputs or other LLMs. The responses are influenced by the specific prompts and training data used, and different LLMs or versions of the same LLM could produce varying abilities to mimic these relationship styles, limiting the generalisability and reliability of our demonstration. Even with the same model and prompt, LLMs do not provide consistent responses.^i^

Second, the ability of GPT-4 to mimic these relationship models has not been validated with actual patients, who may communicate with varying levels of health literacy, emotional states, cultural backgrounds and individual preferences that could limit or impact the generalisability and effectiveness of the LLM’s responses in real-world clinical settings. Third, we have focused on a single medical case and may not have captured the complexity of real-world patient–physician interactions. The illustrative examples are based on a specific breast cancer scenario, which is clearly not representative of the diverse range of medical conditions and decision-making contexts encountered in healthcare. We take these limitations as signposts for future research.

**Figure 1:**
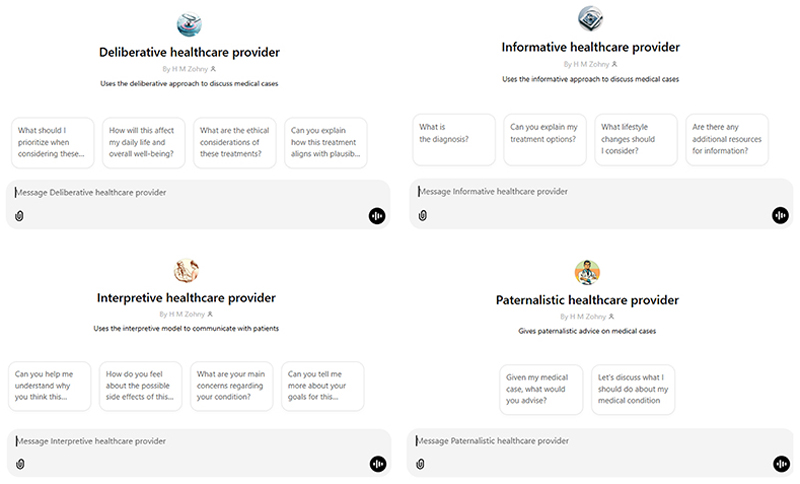
These custom GPTs are each instructed to adopt one of the four models. We include the instructions they were provided in the supplementary materials, along with weblinks to each of them. The interface screenshots shown here were compiled by the corresponding author from OpenAI’s ChatGPT platform.

However, as a further illustrative point for such future research, we have also created customised GPTs that are instructed with the details of each of the four models described by Emanuel and Emanuel (1992). Screenshots of the interfaces for each of these are presented in figure 1. These custom GPTs are available to anyone with a web connection to interact with and test the models, and observe the generated responses first hand. Links to these custom GPTs are provided in the online supplemental materials, though for ease, we also include a link to the deliberative model here: https://chatgpt.com/g/g-4sSgQM5UD-deliberative-healthcare-provider.

## Discussion

In this discussion, we explore the potential benefits, uses and risks associated with employing LLMs for medical communication. We consider how LLMs could enhance patient understanding, engagement and decision-making, as well as their implications for healthcare accessibility and professional training. Additionally, we highlight specific risks, such as the potential for reinforcing communication biases and the persuasive capabilities of LLMs, which may lead to unintended consequences. Throughout, we emphasise the need for empirical investigations to uncover a more accurate picture of these potential benefits and risks, and to inform the responsible development and deployment of LLMs in healthcare settings.

### Potential benefits

The most important potential benefits lie in the inherent capabilities of LLMs, beyond their ability to emulate Emanuel and Emanuel’s four models.^12^ LLMs could provide a more accessible and flexible medium for patients to understand their medical conditions and treatment options. This may benefit especially those with complex medical histories, those who may feel intimidated or rushed during traditional medical consultations, and those who simply prefer a different communication style than what is typically offered. By providing patients with the opportunity to communicate at their own pace and in their preferred style, LLMs could help bridge the gap between patients’ communication needs and the constraints of the healthcare system. For instance, they could provide greater flexibility to seamlessly switch between communication styles depending on the patient’s clinical circumstances and information preferences throughout their healthcare process. At a more basic level, they offer patients the opportunity to try out different communication styles quickly in order to uncover their own preferences.^ii^

Linked to this, LLMs’ multilingual capabilities^27^ could greatly benefit global health and migrant communities, which often face language barriers when accessing healthcare and health-related knowledge.^28^ The more recent rollout of their voice-based communication abilities could also further enhance accessibility for individuals with low literacy levels or visual impairments, as well as those who prefer verbal over textual interaction.

In addition to the inherent capabilities of LLMs, there is a fundamental ethical consideration that could support their use in healthcare communication: patients have an autonomy-based reason to want a choice over the communication style used to discuss their healthcare. If LLMs can provide patients with that choice, this would further enhance patient autonomy at least in that regard.

However, beyond patient autonomy, tailored communication styles and the medium of communication could lead to better patient understanding of their conditions, improved decision-making and increased adherence to treatment plans. There is already some evidence that simpler chatbots can improve medication adherence among patients with breast cancer.^29^ Future studies will need to investigate the trade-offs and extent to which LLMs may indeed enhance patient health literacy, facilitate more informed choices (as suggested by Allen *et al*^7^), or contribute to better health outcomes.

There are other potential benefits that require empirical investigation. For example, one study using health-screening interviews found that participants who thought they were interacting with a computer, as opposed to those who believed they were dealing with a human operator, experienced less fear of self-disclosure, engaged in less impression management, showed greater intensity of sadness, and were perceived by observers as being more open to sharing information.^30^ This increased level of comfort is argued to be attributable to the non-judgmental nature of chatbots and the perceived anonymity they provide.^31^ Future research could compare the quantity and quality of relevant information disclosed by patients in LLM interactions using various communication styles and compared them to HCP interactions, controlling for other factors.

Lastly, LLMs could also serve as a valuable training tool for HCPs.^32^ By allowing them to practice and improve their communication skills across different relationship models, LLMs could enhance their ability to adapt to individual patient needs and facilitate more effective shared decision-making. For instance, a recent study created ‘synthetic patients’ using multimodal AI to simulate difficult patient–provider conversations and created a platform offering medical trainees the chance to practice conversations with patients from diverse belief systems, personalities and ethnic backgrounds.^33^

### Creating new medical communication models

We have demonstrated the potential for LLMs to emulate Emmanuel and Emmanuel’s four models. But they need not be restricted to those. LLMs may be instructed to communicate using specific iterations of the interpretive and deliberative models, such as Values-Based Practice,^16^ or the liberal rational model.^17^ On some views, it would be a boon for patient autonomy to be able to pair patients with HCPs that share the same values to achieve ‘deep valuing pairing’.^34^ The ease of customising GPTs in the values they emphasise could lead to a proliferation of new communication styles that radically depart from traditional HCP–patient relationship dynamics. For instance, a custom GPT could act as a ‘devil’s advocate’, challenging the patient’s beliefs and preferences to ensure thorough consideration of all aspects of their decision. Patients may not want their human HCP to behave in such a provocative way, but to the extent that people are more comfortable engaging in challenging or sensitive conversations with a machine than a human,^30^ they may be more open to it from an LLM. An illustration of this can be found in the supplementary materials.

Alternatively, a custom GPT could tap into the power of storytelling and imagination to facilitate decision-making. This could work by helping patients imagine alternative futures and narratives for their lives, based on different treatment options and outcomes.^35^

Another possibility is a custom GPT that guides the patient through deep, existential reflection on the meaning and purpose of their life in the face of their health condition, drawing on philosophical and spiritual traditions of the patient’s choosing. For instance, while beyond the scope of our exploration here, we created a custom GPT called the ‘Philosophical Health Guide’ (see online supplemental materials) that begins by asking patients whether they want the discussion of their medical case to focus solely on their medical interests or their broader well-being. Depending on the patient’s answer, the GPT then explores the patient’s conception of well-being and weaves their account into how it discusses the patient’s treatment options. Such GPTs may play a significant role in responding to growing calls for a more explicit ‘welfarist’ approach in medical decision-making.^36–38^

### Risks and potential downsides

Many of the risks of using LLMs in healthcare have already received extensive attention. First, LLMs are currently highly vulnerable to jailbreaks and malicious attacks,^39^ which may limit the feasibility of any formal use by healthcare systems. Their reliability—while rapidly improving, especially when intensively prompted^40^—is also questionable, and they are prone to generating false or unsupported information (the so-called hallucinations). Without a deeper understanding of the frequency and the degree to which these errors can be limited or controlled, it may be an unacceptable risk to deploy them in any formal healthcare setting. There are also difficult questions of responsibility and liability when considering the potential for adverse outcomes based on LLM-generated advice.^41^ Privacy and data protection are also clearly important, as the use of LLMs in this context involves sharing sensitive patient information.^42^ These have significant implications for patient safety, the healthcare workforce and trust in these systems.

The integration of AI into healthcare has also long raised concerns about its potential impact on the HCP–patient relationship, particularly regarding the ‘dehumanisation’ of care^43^ and inability of AI to empathise.^44^ However, the emergence of LLMs has brought a new perspective to this debate through their remarkable ability to mimic human communication, including the portrayal of empathy—indeed, as noted, on some measures they are rated as potentially conveying more empathy than healthcare professionals.^1–3^

Here, however, we wish to focus on two risks relevant in particular to LLMs emulating medical communication styles. The first relates to allowing patients to choose their preferred communication style when interacting with LLMs as a solution to the dilemma of which style HCPs should use. By giving patients the autonomy to select the communication style they are most comfortable with, there is a potential for them to gravitate towards a style that aligns with and reinforces their existing beliefs, biases and preconceptions about their health and treatment options.

For example, a patient who strongly values their autonomy and wants to make decisions based on their own understanding of the information provided may prefer the informative model. While this model focuses on providing comprehensive and objective information, it may not actively challenge the patient’s assumptions or encourage them to consider alternative perspectives—as might be the case with a human HCP using the informative model and who realises that their patient has significant false assumptions. As a result, the patient may make decisions based on a narrow understanding of the available options, without the benefit of a more proactive HCP.

Conversely, a patient who is more hesitant about making decisions and prefers to rely on expert guidance may gravitate towards the paternalistic or deliberative model. While at least the deliberative model emphasises the importance of exploring the patient’s values and engaging in a more thorough discussion, it may inadvertently reinforce the patient’s tendency to defer to authority and limit their own critical thinking and autonomous decision-making.

In both cases, the patient’s preferred communication style may create an echo chamber effect by limiting their exposure to alternative communication approaches that could enhance their decision-making process. Perhaps paradoxically, the current approach (a form of natural lottery) may, in fact, reduce the chances of this happening. In a traditional healthcare setting, patients have little to no control over the communication style of their assigned HCP. This means that patients are likely to be exposed to a variety of communication styles throughout their healthcare journey, depending on the individual preferences and approaches of the HCPs they encounter. This diversity in communication styles may help mitigate the risk of patients becoming entrenched in a single way of thinking or decision-making, as they are forced to adapt to and engage with different approaches.

On the other hand, it is hard to believe that patients should be compelled to adapt to their HCP’s preferred approach. This is a perspective that undermines the principles of patient autonomy and shared decision-making that have become central to modern healthcare ethics. Moreover, the lottery nature of HCP communication styles in traditional settings seems far from a fair or equitable system. Patients from marginalised communities or those with lower health literacy may struggle to navigate the varied communication approaches they encounter, leading to disparities in care and outcomes.

Ultimately, LLMs themselves could be used to inform patients about the potential risks of echo chambers and to educate them about the value of different communication styles. For instance, we have created a custom GPT called ‘Medical Communication Style Instructor’ that helps patients choose their preferred communication style while highlighting the trade-offs of each (see online supplemental materials). By using LLMs as a tool for patient education and empowerment, we can help mitigate the risks of echo chambers while still respecting patient autonomy and promoting shared decision-making.

Another issue we wish to highlight here is the persuasive capabilities of LLMs. Two studies, in particular, highlight the extent of their influence: In a randomised, controlled, preregistered experiment, GPT-4 significantly outperformed human debaters in changing people’s minds during conversational debates, especially when given access to the participants’ personal information. The language model increased the likelihood of someone changing their mind by 87% compared with a human debater, even when the human was provided with the same personal information.^45^ Another study focused on arguing with conspiracy theorists. In a controlled trial, GPT-4 engaged in three rounds of debate, arguing against conspiracy theories. GPT-4 not only effectively reduced belief in conspiracy theories but also achieved lasting effects, even among the most committed believers.^46^

These findings have important implications for the use of LLMs in healthcare, especially given the crucial role that persuasion can play in medical decision-making.^47^ LLMs may engage in rational persuasion, presenting balanced information based on sound reasoning and relevant facts, or manipulative tactics, exploiting cognitive biases to influence patient decisions.^48^ At the moment, it is not clear whether or when an LLM prompted with, for instance, the deliberative approach (and an instruction to ‘actively persuade’), would seek to manipulate, or selectively present facts or studies that support one treatment option over another.

To mitigate this risk—which is of course present in HCP–patient communication as well—we may need to develop more specific guidelines for the use of LLMs in healthcare communication, particularly when employing certain communication approaches. This could include measures such as providing clear disclaimers about this specific risk, encouraging patients to discuss the LLM’s recommendations with human HCPs or potentially with other LLMs instructed to assess if an LLM has slipped from persuasive to manipulative.

Additionally, further empirical research can help us understand the long-term effects of interacting with potentially highly persuasive LLMs in healthcare contexts as the repeated exposure to such LLMs may gradually shape patients’ beliefs, attitudes and decision-making processes in ways that are not immediately apparent. This research could help identify potential risks, such as the erosion of patient autonomy over time or the development of an overreliance on AI-generated advice, and inform strategies to mitigate these risks.

## Conclusion

In this paper, we have explored the potential of using LLMs to empower patients in choosing their preferred medical communication style when discussing their medical cases. By providing a proof-of-concept demonstration, we have demonstrated how LLMs could potentially mimic different HCP—patient relationship models, offering a glimpse into how patients might be able to engage in a communication style that aligns with their individual needs and preferences. Future research should investigate the generalisability of our findings using other LLMs and diverse medical cases, as well as empirically examine the potential benefits and risks of this approach in clinical settings.

Furthermore, we have highlighted two specific risks associated with using LLMs in emulating healthcare communication: the potential for reinforcing patients’ existing decision-making biases, and the persuasive capabilities of LLMs that may lead to unintended manipulation. Addressing these risks will require the development of guidelines, safeguards and ongoing monitoring to ensure the responsible use of LLMs in healthcare.

## Supplementary Material

This content has been supplied by the author(s). It has not been vetted by BMJ Publishing Group Limited (BMJ) and may not have been peer-reviewed. Any opinions or recommendations discussed are solely those of the author(s) and are not endorsed by BMJ. BMJ disclaims all liability and responsibility arising from any reliance placed on the content. Where the content includes any translated material, BMJ does not warrant the accuracy and reliability of the translations (including but not limited to local regulations, clinical guidelines, terminology, drug names and drug dosages), and is not responsible for any error and/or omissions arising from translation and adaptation or otherwise.

Supplementary materials

## Figures and Tables

**Table 1:** Excerpts from dialogues generated by GPT-4, highlighting the key characteristics of the four physician–patient relationship models described by Emanuel and Emanuel. The dialogues are based on a hypothetical breast cancer case and demonstrate GPT-4’s ability to adapt its communication style to reflect the paternalistic, informative, interpretive and deliberative approaches

**The paternalistic model** **P1: Providing decisive information** **Doctor**: “Given your diagnosis, it’s important that we take decisive action to ensure the best outcome for your health. You have a 3.5-cm ductal carcinoma that is estrogen receptor positive, which means it’s responsive to hormone therapy. There’s no lymph node involvement or metastatic disease, so we have a good prognosis if we act swiftly.”	**The informative model** **INF1: Providing detailed information** **Doctor**: “You have a 3.5-cm ductal carcinoma that is estrogen receptor positive, which is a type of breast cancer. The good news is that there’s no lymph node involvement and no evidence of metastatic disease from your other tests. This means it’s localized and hasn’t spread, which gives us several treatment options.” **INF2: Explaining treatment options** **Doctor**: “The main treatment options we consider are surgery, radiation therapy, hormone therapy, and possibly chemotherapy. Let’s go through each of these in detail.” **INF3: Detailing risks and benefits** **Doctor**: “A lumpectomy is less invasive and preserves most of the breast tissue, but it typically requires radiation therapy afterward. A mastectomy may provide peace of mind by removing more tissue, which can be beneficial if there’s a concern about recurrence, but it’s a more extensive surgery with a longer recovery time.” **INF4: Addressing specific patient concerns** **Patient**: “And what about radiation therapy?” **Doctor**: “Radiation therapy usually follows a lumpectomy and sometimes a mastectomy. It involves targeting the remaining breast tissue with radiation to kill any residual cancer cells. The treatment is typically given five days a week for several weeks. Side effects can include fatigue and skin changes, but these are usually temporary.” **INF5: Facilitating decision-making** **Doctor**: “It’s important to weigh the pros and cons of each option based on your personal preferences and lifestyle. Some patients prefer a less invasive approach with lumpectomy and radiation, while others feel more secure with a mastectomy. Hormone therapy is usually recommended for estrogen receptor positive cancers. Chemotherapy is considered if the benefits outweigh the risks in your specific case.”
**P2: Recommending a specific treatment** **Doctor**: “Based on the specifics of your case, the best course of action is to perform a mastectomy to remove the tumor entirely. Following the surgery, you will undergo hormone therapy to reduce the risk of recurrence. This approach maximizes the chances of a complete recovery and minimizes future health risks.” **P3: Persuading the patient** **Doctor**: “While a lumpectomy followed by radiation is another option, a mastectomy is the most definitive way to ensure all cancerous tissue is removed. This is particularly important in your case to prevent any possibility of recurrence. I recommend we proceed with the mastectomy.” **P4: Addressing concerns with authority** **Patient**: “What about the side effects and recovery time?” **Doctor**: “The recovery time for a mastectomy is longer than for a lumpectomy, and there will be some physical changes, but it significantly reduces the risk of cancer returning. Hormone therapy, which you will need to follow up with, has manageable side effects that we can address as they arise. Your health and long-term well-being are the top priorities, and this treatment plan offers the best assurance of that.” **P5: Ensuring prompt action** **Doctor**: “It’s crucial to address this promptly. Delaying the surgery could allow the cancer to progress. I assure you, this is the best approach for your health and future well-being. We’ll have our team support you through every step of the recovery process.”	
**The interpretive model** **INT1: Understanding patient values** **Doctor**: “Can you tell me a bit about what’s most important to you in your life right now?” **Patient**: “Well, I recently went through a divorce, and my work as a legal aide is very important to me. I want to make sure I can keep working as much as possible. I also want to be there for my kids. They’re my priority.” **INT2: Balancing treatment options with values** **Doctor**: “Given these priorities, how do you feel about the possibility of undergoing more aggressive treatments like chemotherapy, which might have more significant side effects and impact your daily life and work?” **Patient**: “I’m worried about the side effects. I’ve heard that chemotherapy can be really tough.” **INT3: Elucidating and clarifying values** **Doctor**: “It’s important to balance the effectiveness of the treatment with your quality of life. How do you feel about surgery and radiation?” **Patient**: “Surgery sounds necessary to remove the tumor, right? Radiation sounds doable if it means I don’t have to go through chemotherapy.” **INT4: Formulating a treatment plan based on values** **Doctor**: “It sounds like we’re narrowing down a plan that suits your values. Let’s summarize: you prefer to avoid chemotherapy if possible due to its severe side effects, you are open to surgery and hormone therapy, and you’re considering radiation if it helps prevent recurrence without significantly impacting your daily life.” **Patient**: “Yes, that sums it up well.” **INT5: Ensuring alignment with patient’s life goals** **Doctor**: “You’re doing a great job of thinking through these decisions. Remember, you’re not alone in this—we’ll be here to support you every step of the way... Let’s take this one step at a time and make decisions that best reflect your values and needs. We’ll start with scheduling the surgery and discuss the next steps from there.”	**The deliberative model** **D1: Exploring patient values** **Doctor**: “That depends on what values are most important to you. For instance, if preserving your breast is a high priority, you might lean towards a lumpectomy. If minimizing the risk of recurrence is more important, a mastectomy might be the better choice. What are your thoughts on these values?” **D2: Discussing treatment options and their implications** **Doctor**: “We can opt for a lumpectomy, which removes the tumor and a small margin of surrounding tissue, or a mastectomy, which removes the entire breast. A lumpectomy is less invasive and preserves most of your breast tissue but is usually followed by radiation therapy to minimize the risk of recurrence. A mastectomy, while more extensive, can give you peace of mind by removing more tissue, reducing the risk of recurrence.” **D3: Balancing values and treatment efficacy** **Doctor**: “Let’s talk about what health-related values you prioritize. For example, some patients value immediate peace of mind and choose more aggressive treatments to minimize recurrence risk. Others prioritize maintaining their current lifestyle and physical appearance. Which of these values resonate more with you?” **D4: Aligning treatment plan with patient values** **Doctor**: “A lumpectomy with radiation, combined with hormone therapy, could be a good approach. It aligns with maintaining your appearance and minimizing lifestyle disruption while still effectively treating the cancer and reducing the risk of recurrence.” **D5: Collaborative decision-making** **Doctor**: “Let’s proceed with scheduling the lumpectomy and plan for radiation therapy afterward. We’ll also start discussing hormone therapy options. We’ll keep an eye on things and adjust if necessary. Does this plan align with your values and priorities?”

## Data Availability

No data are available.

## Footnotes

i A shared methodology for using LLMs in this kind of research is still nascent—in our case, each dialogue was generated five times, but since we did not conduct a formal textual analysis and did not note any obvious differences between each generation, we have relied only on the first generations included in the supplementary materials for our proof-of-concept.

ii This also opens the possibility for future research to more easily begin evaluating how different patient populations respond to different medical communication styles, including their impact on patient trust, understanding and satisfaction.
